# The effects of chilling-light stress on photosystems I and II in three *Paphiopedilum* species

**DOI:** 10.1186/s40529-017-0208-4

**Published:** 2017-11-25

**Authors:** Ying-Jie Yang, Wei Chang, Wei Huang, Shi-Bao Zhang, Hong Hu

**Affiliations:** 1 Key Laboratory of Economic Plants and Biotechnology, Kunming Institute of Botany, Chinese Academy of Sciences, 132# Lanhei Road, Heilongtan, Kunming, 650201 Yunnan China; 2 Yunnan Key Laboratory for Wild Plant Resources, 132# Lanhei Road, Heilongtan, Kunming, 650201 Yunnan China; 3 University of Chinese Academy of Sciences, 19 A Yuquan Rd, Shijingshan District, Beijing, 100049 People’s Republic of China

**Keywords:** Chilling temperature, *Paphiopedilum*, Photoinhibition, Photosystem I, Photosystem II, Cyclic electron flow

## Abstract

**Background:**

Low temperatures pose a critical limitation to the physiology and survival of chilling-sensitive plants. One example is the genus *Paphiopedilum* (Orchidaceae), which is mainly native to tropical and subtropical areas from Asia to the Pacific islands. However, little is known about the physiological mechanism(s) underlying its sensitivity to chilling temperature. We examined how chilling-light stress influences the activities of photosystem I (PSI) and photosystem II (PSII) in three species: *P. armeniacum*, *P. micranthum*, and *P. purpuratum*. All originate from different distribution zones that cover a range of temperatures.

**Results:**

Photosystem II of three *Paphiopedilum* species was remarkable sensitivity to chilling stress. After 8 h chilling stress, the maximum quantum yield of PSII of three species of *Paphiopedilum* was significantly decreased, especially in *P. purpuratum*. The quantity of efficient PSI complex (*P*_*m*_) value did not significantly differ after 8 h chilling treatment compared to the original value in three species. The stronger PSII photoinhibition and significantly less capacity for cyclic electron flow (CEF) were observed in *P. purpuratum.*

**Conclusions:**

In conclusion, the three species of *Paphiopedilum* showed significant PSII photoinhibition when exposed to 4 °C chilling treatment. However, their PSI activities were not susceptible to chilling-light stress during 8 h. The CEF was important for the photoprotection of PSI and PSII in *P. armeniacum* and *P. micranthum* under chilling conditions. Our findings suggested that the photosynthetic characteristics of *Paphiopedilum* were well adapted to their habitat.

## Background

The activities associated with energy capture and electron transfer are essential for photosynthesis. These reactions perform high-potential redox chemistry and lead to photodamage of the photosynthetic machinery. Photosynthesis can be very susceptible to many suboptimal environmental conditions, e.g., chilling temperatures, particularly in plants of tropical or sub-tropical origin (Havaux and Davaud 1994; Sonoike 1995; Zhang and Scheller 2004; Huang et al. 2010a, b). Members of *Paphiopedilum* are ornamental plants prized for their large, slipper-shaped floral labellums. Because of this, over-collection of the genus has become so extensive that many species are now sub-viable in their natural habitats. These plants usually occur in limestone or mountainous forests within tropical and subtropical zones ranging from Asia to the Pacific islands (Cribb 1998). Due to their tropical or subtropical origins, it is believed that these orchids cannot survive in regions with long-term natural chilling temperatures. However, the mechanism underlying their potential adaption to such conditions is unknown.

Generally, the decline in photosynthesis activity in response to chilling is attributed to a depression of Rubisco activity and RuBP regeneration, which consequently restricts the rate of CO_2_ assimilation (Bernacchi et al. 2003; Sage and Kubien 2007). Such stress can disrupt all of the key processes of photosynthesis, including thylakoid electron transport, the carbon reduction cycle, and control of stomatal conductance (Damian and Donald 2001). Because photochemical efficiency is suppressed by cold temperatures, the level of absorbed light energy exceeds that required for photosynthesis. If this excess energy cannot be dissipated as heat via non-photochemical quenching (NPQ) in a timely manner, a large amount of reactive oxygen species (ROS) is formed in the cells (Asada 1999).

The PSII reaction center is thought to be damaged when the structure of relevant protein components is destroyed (Barber and Andersson 1992; Aro et al. 1993; Havaux and Davaud 1994). However, in a recently introduced two-step scheme for PSII photoinhibition, it is now assumed that ROS does not directly induce oxidative damage to the PSII functional center but, instead, exacerbates this photoinhibition by suppressing the synthesis of proteins, notably the D1 protein, which has a vital role in the repair process for PSII (Nishiyama et al. 2001, 2004, 2011; Murata et al. 2007; Takahashi et al. 2009). Normally, plants have antioxidant systems, e.g., the water–water cycle, which eliminate ROS. However, under stress conditions such as chilling, excess ROS can accumulate and cause a cellular imbalance, ultimately leading to damage to photosynthetic apparatus (Asada 1999). In chilling-sensitive plants, such as cucumber (*Cucumis sativus*) and *Arabidopsis thaliana*, PSII photoinhibition is negligible during short-term treatment with chilling-light stress (Sonoike 1995; Zhang and Scheller 2004). In tropical tree species, however, PSII is selectively damaged when plants are exposed to chilling temperature associated with moderate light intensity (Huang et al. 2010a, b). Therefore, the response of PSII activity to cold temperatures differs among chilling-sensitive species. Furthermore, the effect of such stress on PSII activity in *Paphiopedilum* species is unclear.

Because low temperatures depress the rate of CO_2_ assimilation, active electrons being transported through linear electron flow from PSII are accumulated at PSI, a phenomenon that contributes to the generation of hydroxyl radicals and over-reduction on the acceptor side in PSI (Sonoike 2006). As a result, the PSI acceptor side is attacked by oxidative hydroxyl radicals. Whereas PSI in sensitive cucumber and *Arabidopsis* is selectively damaged at chilling temperatures associated with moderate light intensity (Havaux and Davaud 1994; Terashima et al. 1994; Zhang and Scheller 2004), PSI activity in tropical trees is not susceptible to that stress combination (Huang et al. 2010a, b). Therefore, the performance of PSI shows obvious differences among species. Severe, irreversible photodamage to PSI activity can subsequently lead to PSII photoinhibition and even plant death (Suorsa et al. 2012). It remains unclear whether PSI activity in *Paphiopedilum* species is sensitive to chilling-light stress.

Here, we examined the influence of combined chilling and light stress on PSI and PSII activities in three species of *Paphiopedilum* that have different habitat zones of distribution. We postulated that, under stress conditions, species distributed in a tropical region would show stronger photoinhibition of PSI and/or PSII than species from a subtropical area.

## Methods

### Plant materials

Three species of *Paphiopedilum* were chosen, including *P. armeniacum*, which mainly occurs in the southwestern portion of Yunnan Province. This is one of the few species within the genus that can grow at elevations above 1500 m. Another species, *P. micranthum*, has a relatively wide-spread distribution, primarily in the subtropical regions of China, while *P. purpuratum* is found below 1000 m and only in the tropical regions of southernmost China and Vietnam. Before our trials began, all plant materials were cultivated for optimal growth in a greenhouse at the Kunming Institute of Botany (102º41′E, 25º01′N). Conditions included 20–25 °C and a photosynthetic photon flux density (PPFD) of approximately 15–20% of full sunlight.

### Chilling treatment

Mature leaves collected from 12 healthy plants per species were exposed to 4 °C for 8 h in a cool room. For each species, six of the 12 detached leaves were placed on wet paper under a PPFD of 200 μmol photons m^−2^ s^−1^ while the other six were tested at 500 μmol photons m^−2^ s^−1^. To determine the response of PSI and PSII to the chilling stress, detached leaves incubated in the presence or absence of lincomycin (1 mM) overnight at 25 °C in darkness were transferred to 4 °C and 500 μmol photons m^−2^ s^−1^ for 8 h. Relevant physiological measurements were conducted before and during the treatment period.

The 8 h chilling treatment was conducted in one phytotron at 4 ± 1 °C (chilling temperature). Twelve healthy plants per species were used. For leaves of both species, light intensity was controlled at 250–300 µmol photons m^−2^ s^−1^ for 8 h. Other experimental conditions included 60% relative humidity and a CO_2_ concentration of 400 µmol mol^−1^. Chlorophyll fluorescence and P700 parameters were recorded.

### Chlorophyll fluorescence and P700 measurements

The chlorophyll fluorescence of PSII was measured with a Dual PAM-100 system (Heinz Walz, Effeltrich, Germany) connected to a computer with control software. To develop light response curves, mature leaves were illuminated at 200 μmol photons m^−2^ s^−1^ for at least 20 min at 20 °C. Light-adapted photosynthetic parameters were then recorded after exposure to each light for 2 min. The following parameters were calculated: *F*_*v*_/*F*_*m*_ = (*F*_*m*_ − *F*_*o*_)/*F*_*m*_, Y(II) = (*F*_*m*_′ − *F*_*s*_)/*F*_*m*_′ (Genty et al. 1989), and NPQ = (*F*_*m*_ − *F*_*m*_′)/*F*_*m*_′ (Bilger and Björkman 1991). Here, *F*_*v*_/*F*_*m*_ was the maximum quantum yield of PSII after 20 min dark adaptation; *F*_*v*_ represented variable chlorophyll fluorescence; *F*_*o*_ was the minimum fluorescence in the dark-adapted state (20 min); *F*_*m*_ and *F*_*m*_′ were the dark-adapted and light-adapted maximum fluorescence upon illumination with a pulse (300 ms) of saturating light (10,000 μmol photons m^−2^ s^−1^), respectively; Y(II) was the effective quantum yield of PSII photochemistry; and *F*_*s*_ was the light-adapted steady-state fluorescence. The fraction of energy passively dissipated in the form of heat and fluorescence, Y(NO) = *F*_*s*_ */F*_*m*_ (Hendrickson et al. 2004; Kramer et al. 2004), *F*_*s*_, light-adapted steady state fluorescence. The fraction of energy dissipated in the form of heat via the regulated non-photochemical quenching mechanism, Y(NPQ) = *F*_*s*_ */F*_*m*_′ − *F*_*s*_ */F*_*m*_ (Kramer et al. 2004).

Simultaneously, the maximum photo-oxidizable P700 (*P*_*m*_) were determined with saturation pulses using the Dual PAM-100 system. This variable represents the maximum alteration in signal upon quantitative transformation of P700 from the fully reduced to the fully oxidized state. At a defined optical property, the amplitude of *P*_*m*_ depends on the maximum amount of photo-oxidizable P700, which is a good parameter for reflecting the quantity of efficient PSI complex (Huang et al. 2010a, b, 2013). Leaves were dark-adapted for 20 min before *P*_*m*_ measurement (Huang et al. 2010a, b; Suorsa et al. 2012; Tikkanen et al. 2014). After far-red pre-illumination for 10 s, *P*_*m*_ was determined by applying a saturation pulse (Klüghammer and Schreiber 2008). *P*_*m*_′ was determined similarly, except that background actinic light was used instead of far-red illumination. The photochemical quantum yield of PSI, Y(I), is defined by the fraction of overall P700 that, in a given state, is reduced and not limited by the acceptor side (Pfundel et al. 2008). We used Dual-PAM-100 software to calculate Y(I) = (*P*_*m*_′ − *P*)/*P*_*m*_. Parameter Y(ND) is estimated as P/Pm and represents the fraction of overall P700 that is oxidized in a given state that is enhanced by a trans-thylakoid proton gradient and photodamage to PSII. Y(NA) represents the fraction of overall saturation P700 that cannot be oxidized by a saturation pulse in a given state due to a lack of acceptors, which was calculated as (*P*_*m*_ − *P*_*m*_′)*/P*_*m*_.

Photosynthetic electron flows through PSI and PSII were computed as: ETRII = Y(II) × PPFD × abs I × 0.5 and ETRI = Y(I) × PPFD × abs I × 0.5. Here, 0.5 was the proportion of absorbed light reaching PSI or PSII, and abs I was the absorbed irradiance set to 0.84 of incident irradiance. We estimated cyclic electron flow around PSI (CEF) as the difference in electron flow between PSI and PSII (Miyake et al. 2005; Huang et al. 2012).

### Statistical analysis

Statistical analysis was performed with SPSS 16.0. Data were subjected to analysis of variance (ANOVA), and Tukey’s multiple comparison tests were used to determine whether significant differences existed between treatments at α = 0.05.

## Results

**Fig. 1:**
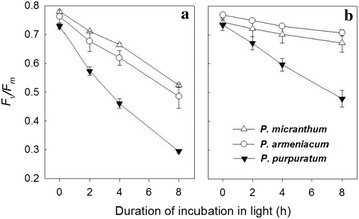
Changes in *F*_*v*_ */F*_*m*_ in three species of *Paphiopedilum* during chilling treatment at 4 °C under photosynthetic flux densities of 500 (**a**) or 200 (**b**) μmol photons m^−2^ s^−1^. Mean ± SE was calculated from six independent plants per species

Before the chilling-light treatment, the maximum quantum yield of PSII did not differ significantly among species. As the experiment proceeded, all of the tested leaves showed a decline in *F*_*v*_/*F*_*m*_, but the amplitudes differed (Fig. 1). For example, at a relatively low level of 200 μmol photons m^−2^ s^−1^, photoinhibition of PSII was stronger in *P. purpuratum* than in *P. armeniacum* and *P. micranthum*. When exposed to the chilling temperature for 8 h, *F*_*v*_/*F*_*m*_ from *P. armeniacum* and *P. micranthum* decreased slightly, from 0.75 to 0.70, while the value for that parameter dropped from 0.73 to 0.49 in *P. purpuratum*. When exposed to a strong light of 500 μmol photons m^−2^ s^−1^, PSII photoinhibition was remarkably aggravated, with rapid *F*_*v*_/*F*_*m*_ decreases of 36, 32, and 59% in *P. armeniacum*, *P. micranthum*, and *P. purpuratum*, respectively.

**Fig. 2:**
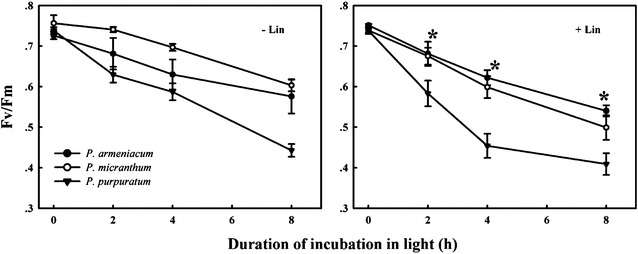
Change in *F*_*v*_ */F*_*m*_ in the three *Paphiopedilum* species during chilling treatment at 4 °C under photosynthetic flux densities of 500 μmol photons m^−2^ s^−1^ in the presence and absence of lincomycin. Mean ± SE was calculated from six independent plants per species

**Fig. 3:**
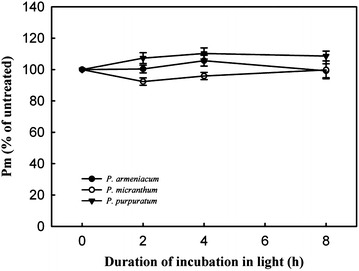
Maximum photo-oxidizable P700 (*P*_*m*_) during chilling treatment at 4 °C under photosynthetic flux densities of 500 μmol photons m^−2^ s^−1^ in three species of *Paphiopedilum.* Mean ± SE was calculated from six independent plants per species

In order to determine whether the rate of PSII photodamage at chilling-light stress differs between the three species, detached leaves were pre-incubated with lincomycin and exposed to 500 μmol photons m^−2^ s^−1^. Lincomycin, an inhibitor of chloroplast translation machinery, prevents the de novo synthesis of D1 protein and thus stops the turnover of PSII. The presence of Lincomycin made it possible to evaluate the rate of PSII photodamage. During chilling treatment at 4 °C and 500 μmol m^−2^ s^−1^ without lincomycin, both species showed the same extent of decrease in F_v_/F_m_ (Fig. 2 −Lin). However, with pre-treatment by lincomycin, *P. purpuratum* showed significantly decrease in F_v_/F_m_ than the other species (Fig. 2 +Lin), indicating that the rate of PSII photodamage at this chilling-light stress was greater in *P. purpuratum*. During 8 h treatment, no photoinhibition of PSI was observed in any of these species, with *P*_*m*_ values did not significantly differ compared to the original value in three species (Fig. 3), suggesting that PSI was insusceptible to chilling-light stress in three species.

**Fig. 4:**
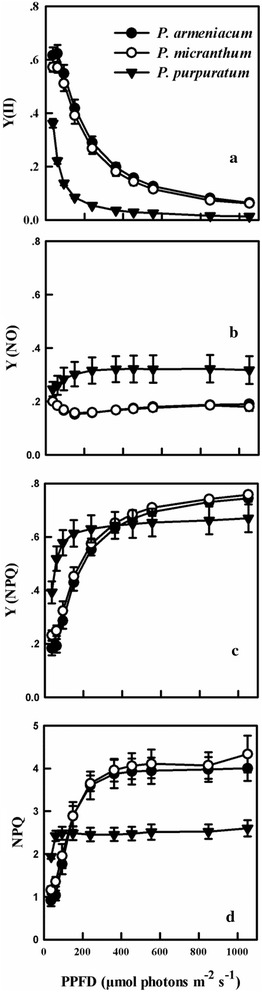
Light response curves for Y(II) (**a**); Y(NO) (**b**); Y(NPQ) (**c**) and NPQ (**d**) from three species of *Paphiopedilum* at 25 °C. Mean ± SE was calculated from six independent plants per species

**Fig. 5:**
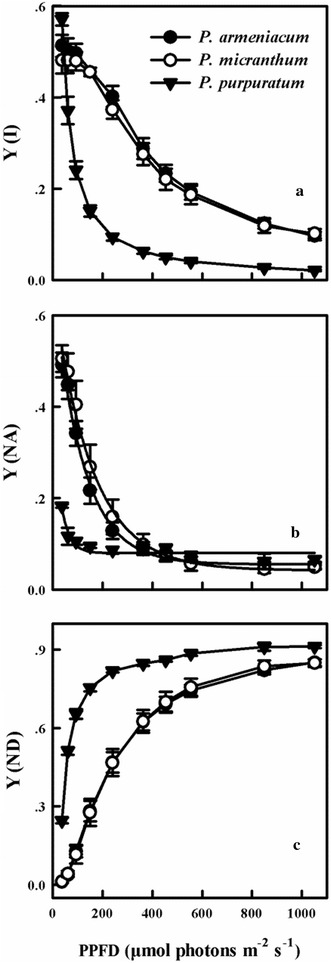
Light response curves for Y(I) (**a**); Y(NA) (**b**) and Y(ND) (**c**) from three species of *Paphiopedilum* under 25 °C. Mean ± SE was calculated from six independent plants per species

Before chilling treatment, the light response curves of three *Paphiopedilum* species indicated that both the capacity for effective quantum yield of PSII [Y(II)] and non-photochemical quenching (NPQ) were significantly lower in *P. purpuratum* (Fig. 4a, d). The quantum yield of non-regulated energy dissipation [Y(NO)] was significantly higher in *P. purpuratum* than in the other species and gradually increased with PPFDs increased (Fig. 4b). Y(NPQ) and NPQ were increased at low PPFDs in three species (Fig. 4c, d), but significantly lower in *P. purpuratum* than in the other species. The effective quantum yield of PSI [Y(I)] and Y(NA) were significantly lower, but Y (ND) was significantly higher in *P. purpuratum* than in the other species (Fig. 5). These results showed that, compared with the other two species, *P. purpuratum* not only had a lower ability to utilize light energy, but also displayed less capacity to dissipate excess energy harmlessly as heat. Therefore, it was difficult to detect the light response curve of *P. purpuratum* after 8 h chilling treatment and the results did not provide the valid and believable data. The value of CEF at 200 μmol photons m^−2^ s^−1^ was detected to compare the CEF of three species after chilling treatment.

**Fig. 6:**
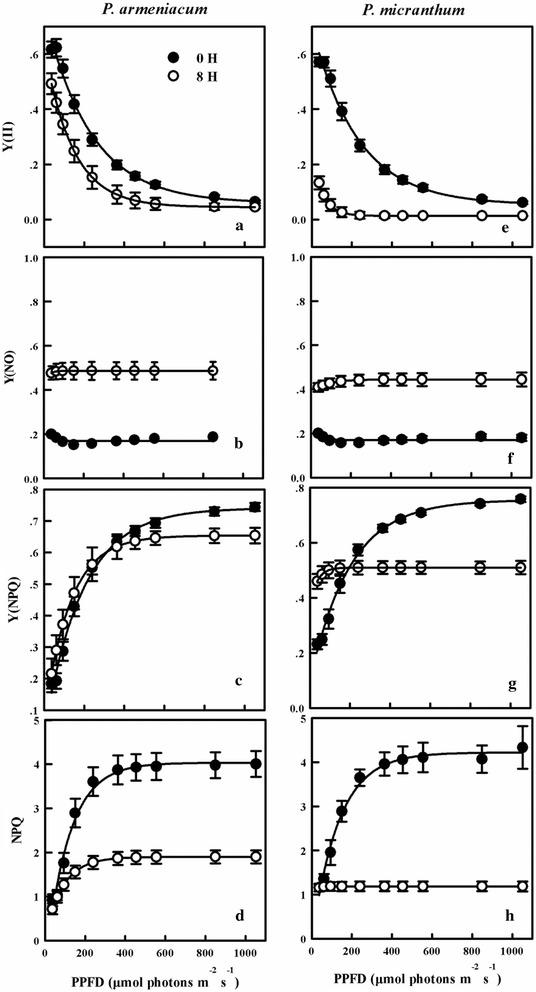
Light response curves for Y(II) (**a**, **e**); Y(NO) (**b**, **f**); Y(NPQ) (**c**, **g**) and NPQ (**d**, **h**) from three species of *Paphiopedilum* after 8 h chilling stress. Mean ± SE was calculated from six independent plants per species

Compared with the plant untreated, values of Y(II) significantly decreased after chilling treatment and the values of *P. micranthum* was significantly lower than *P. armeniacum* (Fig. 6a, e). Y(NPQ) increased at low PPFDs in the two species (Fig. 6c, g) and significantly decreased with PPFDs increased. Values of NPQ of the two species were significantly lower than untreated plants, whereas Y(NO) of the two species were significantly higher than untreated plants (Fig. 6b, d, f, h).

**Fig. 7:**
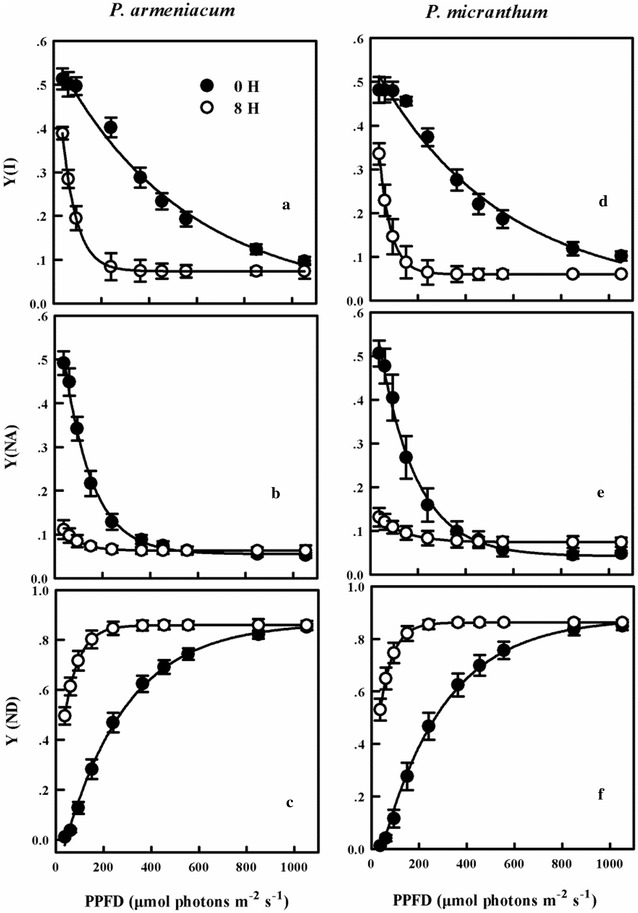
Light response curves for Y(I) (**a**, **d**); Y(NA) (**b**, **e**) and Y(ND) (**c**, **f**) from three species of *Paphiopedilum* after 8 h chilling stress. Mean ± SE was calculated from six independent plants per species

The values of Y(I) of *P. armeniacum* and *P. micranthum* significantly decreased after chilling treatment (Fig. 7a, d). For the two species, Y(NA) was significantly decreased under low light intensity, however little increased under higher light intensity (Fig. 7b, e). Compared with untreated status, the values of Y(ND) significantly increased after chilling treatment (Fig. 7c, f).

**Fig. 8:**
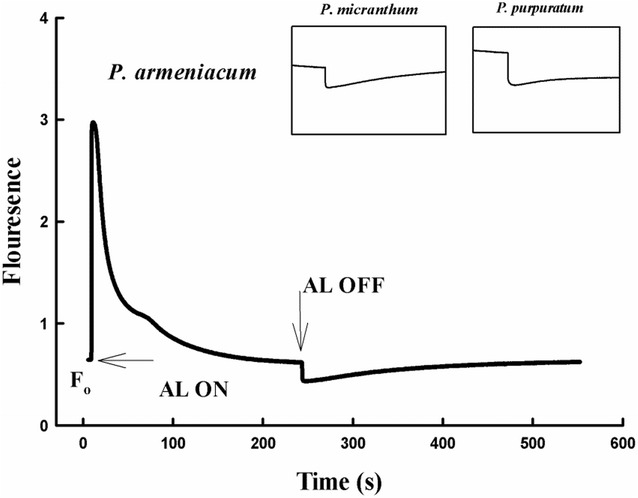
Monitoring of NDH activity by chlorophyll fluorescence. Mature leaves of three *Paphiopedilum* species were exposed to actinic light (AL, 200 μmol photons m^−2^ s^−1^) after the measuring light was turned on (Fo, the minimum level of chlorophyll fluorescence). The AL was turned off and the subsequent change in chlorophyll fluorescence was monitored as an indicator of NDH activity. The mature leaf was dark-adapted at 25 °C for 30 min, and the chlorophyll fluorescence was measured at 25 °C

**Fig. 9:**
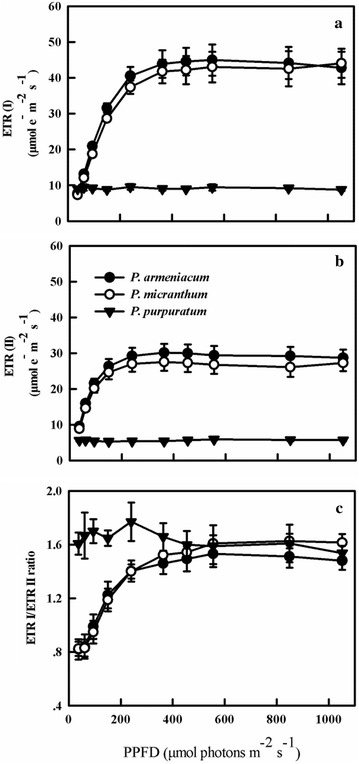
Light-response changes in photosynthetic electron flow through PSI (ETRI) (**a**) and PSII (ETRII) (**b**), and ETRI/ETRII ratios (**c**) in leaves from three species of *Paphiopedilum* at 25 °C. Mean ± SE was calculated from 6 independent plants per species

**Fig. 10:**
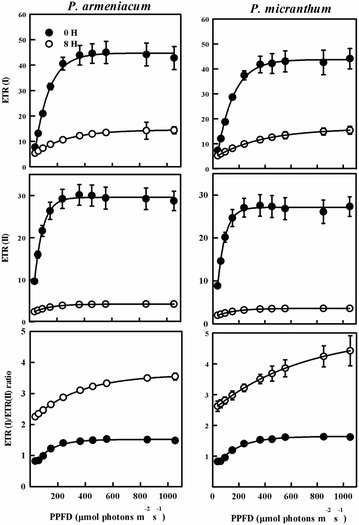
Light-response changes in photosynthetic electron flow through PSI (ETRI) and PSII (ETRII), and ETRI/ETRII ratios in leaves from two species of *Paphiopedilum* after 8 h chilling stress. Mean ± SE was calculated from six independent plants per species

**Fig. 11:**
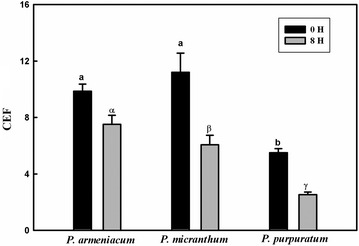
The cyclic electron flow around PSI (CEF) at 200 μmol photons m^−2^ s^−1^ in three *Paphiopedilum* species after 8 h chilling stress. Each vertical bar represents Mean ± SE for four measurements from individual plants. Different letters above bars indicate significant differences between treatment (P < 0.005, based on ANOVA, followed by Tukey’s post hoc tests for comparison)

Two cyclic pathways around PSI have been identified in C3 plants; the first pathway is NDH pathway that is able to reduce plastoquinones from stromal NAD(P)H donors (Horvath et al. 2000) and the second pathway is PGR5 pathway, which is localized in the chloroplast and considered to be a factor for major cyclic electron transport activity in C3 plants (Munekage et al. 2002, 2004, 2008). The results indicated the NDH pathway in three species of *Paphiopedilum* (Fig. 8). Before chilling treatment, ETR(I) and ETR(II) of *P. purpuratum* was significantly lower than the other species. The ETRI/ETRII ratio can serve as an indicator of CEF activation (Yamori et al. 2011). Our data also showed that ETR(I)/ETR(II) ratio continued to increase when the light intensity changed from 0 to 400 μmol photon m^−2^ s^−1^, with final values being 1.6 for *P. micranthum* and 1.4 for *P. armeniacum*. By contrast, the ETRI/ETRII ratio for *P. purpuratum* approximately remained stable at 1.6 under tested light intensity (Fig. 9). Furthermore, Y (ND) of three species was high under high light which indicated the presence of PGR5 pathway (Fig. 5). After chilling treatment, *P. armeniacum* and *P. micranthum* showed significantly lower ETR(I) and ETR(II) but significantly higher ETR(I)/ETR(II) ratio. *P. armeniacum* had higher values of the ETR(I)/ETR(II) ratio than *P. micranthum* (Fig. 10). After chilling treatment, the values of CEF at 200 μmol photons m^−2^ s^−1^ in *P. purpuratum* were significantly lower than the other species (Fig. 11). These results demonstrated that CEF activation had occurred in each species and albeit to different degrees.

## Discussion

*Paphiopedilum* species are very well-known slipper orchids in horticulture and endangered. The genus *Paphiopedilum* occurs mainly in tropical and subtropical of Asia, and the most northerly distributed species occur in Yunnan and Guizhou province which must endure chilling-light stress in winter. However, the responses of PSI and PSII in *Paphiopedilum* to chilling-light stress are little known.

In our present study, the maximum quantum yield of PSII after dark adaptation was significantly decreased in three species of *Paphiopedilum* especially in *P. purpuratum* indicated that chilling-light stress caused significantly PSII photoinhibition in three species of *Paphiopedilum*, especially in *P. purpuratum*. This photoinhibition is a net result of photodamage and repair, stronger photoinhibition indicates that the rate of PSII photodamage exceeds that of repair. This repair of the photo-damaged PSII complex is mainly dependent on ATP synthesis (Allakhverdiev et al. 2005). At chilling temperatures, photosynthetic electron transport from PSII to NADP^+^ is blocked because of several mechanisms, including inhibition of the Calvin–Benson Cycle and depression of biochemical reactions (Murata et al. 2007). Consequently, the rate of PSII repair is very low under cold conditions (Allakhverdiev and Murata 2004). Furthermore, repair of that complex is based on new synthesis of D1 protein, which can be deterred by ROS (Nishiyama et al. 2001, 2004, 2011). At a chilling temperature, inhibition of CO_2_ assimilation accelerates ROS production (Takahashi et al. 2007). Our data showed that the decrease in *F*_*v*_ */F*_*m*_ was linearly over time in all three species, especially for leaves of *P. purpuratum* and in the presence of lincomycin, thereby suggesting that PSII repair was strongly inhibited by the reduced temperature. Before chilling treatment, *P. purpuratum* had significantly lower NPQ and higher Y(NO) than the other species also indicated the PSII photodamage (Takahashi et al. 2009). After chilling treatment, the values of Y(NO) significantly increased in *P. armeniacum* and *P. micranthum*, while the values of NPQ and Y(NPQ) of *P. micranthum* were strongly stimulated and significantly lower than *P. armeniacum*. These results suggested that excess light energy could not be consumed through photochemical quenching and NPQ. This indicated that the PSII reaction center activities were strongly down-regulated or reversibly damaged by the chilling stress. It is concluded that the photoinhibition of PSII in *Paphiopedilum* species was caused by a high level of non-regulated energy dissipation which was induced by 8 h chilling stress.

The rate of PSII photodamage is mainly correlated with CEF-dependent generation of ∆pH (Takahashi et al. 2009; Huang et al. 2012) and the production of ROS (Oguchi et al. 2009, 2011). For example, as light levels increase, PRG5-dependent CEF generates strong ∆pH by blocking proton transport from the lumen back to the stroma via ATP synthase (Tikkanen et al. 2014). The CEF-dependent generation of ∆pH can drive a Ca^2+^/H^+^ antiport to sequester Ca^2+^ in the lumen, which favors the stabilization of the oxygen-evolving complex (OEC) of PSII, where most of the photodamage occurs. Inactivation of the OEC can enhance the level of P680^+^, subsequently damaging the PSII reaction centers (Takahashi and Murata 2008). Thus, impairment of PRG5-dependent CEF activity accelerates photodamage to both the OEC and PSII activity (Takahashi et al. 2009). Our results indicated that *P. purpuratum* had significantly less CEF capacity than the other species at 200 μmol photos m^−2^ s^−1^, which was consistent with the tendency of PSII in our study. We therefore concluded that CEF played an important role in protecting PSII from photoinhibition under temporal chilling in the three species. In addition, the more chilling-sensitive of *P. purpuratum* showed lower NPQ capacity. Because the role of NPQ is to diminish ROS formation, we would expect the rate of production to be greater in *P. purpuratum* under chilling stress. Although the role of ROS in accelerating PSII photodamage remains controversial, Oguchi et al. (2009, 2011) have indicated that ROS can cause oxidative damage to PSII under high light. Therefore, the reduced NPQ capacity in *P. purpuratum* was probably another important mechanism that made its PSII more susceptible to chilling-light stress when compared with *P. micranthum* and *P. armeniacum*.

The sensitivity of PSI to chilling-light stress varies among species. Some chilling-sensitive species are more vulnerable to low temperature, including cucumber and *Arabidopsis*, display significant PSI photoinhibition after such combined stress treatments (Sonoike 1995, 1996; Sonoike et al. 1995; Zhang and Scheller 2004). However, PSI activity is not susceptible to chilling-light in other plants, such as tropical tree species and two *Cymbidium* species (Huang et al. 2010a, b, Li and Zhang 2016). We demonstrated that the quantity of efficient PSI complex, or *P*_*m*_, remained relatively stable in all three species during the experimental period. After 8 h chilling treatment, the PSI activity of *P. armeniacum* and *P. micranthum* had the same varying trend.

Photoinhibition of PSI can occur under two scenarios: (1) over-reduction of the PSI acceptor side and (2) electron transport from PSII to PSI (Sonoike 2006, 2011). Strong illumination is, in principle, very harmful to PSI if the amount of electrons fed to the electron transfer chain by PSII exceeds the capacity of electron acceptors on the reducing side of PSI (Tikkanen et al. 2014). Indeed, PSI utilizes opposing strategies to cope with photo-oxidative stress. For example, the PGR5-dependent CEF pathway is essential for the photoprotection of PSI because it increases the fraction of oxidized P700 and prevents over-reduction of the acceptor side (Munekage et al. 2002, 2004). In *P. micranthum* and *P. armeniacum*, higher CEF activity prevented this over-reduction and minimized PSI photodamage whereas CEF activity was markedly lower in *P. purpuratum*. The higher values of Y(ND) and lower values of Y(NA) of *P. armeniacum* and *P. micranthum* also indicated that PSI activity was being protected from chilling photodamage which was favored the survival of the two species during winter.

When electron transfer to PSI from PSII is strictly controlled, for example, in the presence of DCMU, PSI not only is protected against photo-oxidative stress (Sonoike 1996), but can also function as an extremely efficient quencher of excitation energy captured by the light harvesting machinery (Tikkanen et al. 2014). We showed that *P. purpuratum* had much lower values for ETR(II) under tested light intensities. This led to a high level of photo-oxidizable P700 and protected PSI from permanent photodamage. In addition, PSII photoinhibition is the ultimate regulator of photosynthetic electron flow and provides a photoprotective mechanism against damage to PSI (Tikkanen et al. 2014). Here, after 8 h of chilling at 500 μmol photons m^−2^ s^−1^, *F*_*v*_/*F*_*m*_ decreased by 59% in *P. purpuratum*. The severe PSII photoinhibition in that species was largely responsible for preventing excess electron flow to PSI, thereby allowing the amount of active PSII to be balanced with the capacity of the PSI electron acceptors. We assumed that the lack of susceptibility by PSI activity to chilling-light stress in *P. purpuratum* was probably not due to CEF activation but rather to the inhibition of electron transport from PSII to PSI.

In conclusion, the three species of *Paphiopedilum* showed significant PSII photoinhibition when exposed to 4 °C chilling treatment. However, their PSI activities were not susceptible to chilling-light stress during 8 h. The relative higher CEF activity in *P. armeniacum* and *P. micranthum* alleviated PSII photoinhibition and protected PSI activity in stressed leaves. In the most sensitive species, *P. purpuratum*, lower CEF activity led to severe chilling-induced PSII photoinhibition. Compared with the other two species, greater CEF capacity in *P. armeniacum* alleviated chilling-induced PSII photoinhibition, which is correlated with its higher elevation distribution. Our findings suggested that the photosynthetic characteristics of *Paphiopedilum* were well adapted to their habitat.

## Abbreviations

PSI: photosystem I
PSII: photosystem II
*F*_*v*_*/F*_*m*_: the maximum quantum yield of PSII
*P*_*m*_: the maximum photo-oxidizable P700
CEF: cyclic electron flow
NPQ: non-photochemical quenching
PPFD: photosynthetic photon flux density
*F*_*0*_: the minimum fluorescence in the dark-adapted state
*F*_*m*_: the dark-adapted maximum fluorescence upon illumination with a pulse (300 ms) of saturating light (10,000 μmol photons m^−2^ s^−1^)
*F*_*m*_′: the light-adapted maximum fluorescence upon illumination with a pulse (300 ms) of saturating light (10,000 μmol photons m^−2^ s^−1^)
*F*_*s*_: light-adapted steady state fluorescence
*F*_*s*_: the light-adapted steady-state fluorescence
Y(II): the effective quantum yield of PSII photochemistry
Y(NO): the light-adapted steady state fluorescence
Y(NPQ): the fraction of energy dissipated in the form of heat via the regulated non-photochemical quenching mechanism
*Pm*′: the maximum change in P700 in a given light state
Y(I): quantum yield of PSI
Y(ND): the fraction of overall P700 that is oxidized in a given state that is enhanced by a trans-thylakoid proton gradient and photodamage to PSII
Y(NA): the fraction of overall saturation P700 that cannot be oxidized by a saturation pulse in a given state due to a lack of acceptors
ETRI: photosynthetic electron flow through PSI
ETRII: photosynthetic electron flow through PSII
abs I: the absorbed irradiance set to 0.84 of incident irradiance
OEC: the oxygen-evolving complex
